# The Solar-Induced Chlorophyll Fluorescence Imaging Spectrometer (SIFIS) Onboard the First Terrestrial Ecosystem Carbon Inventory Satellite (TECIS-1): Specifications and Prospects

**DOI:** 10.3390/s20030815

**Published:** 2020-02-03

**Authors:** Shanshan Du, Liangyun Liu, Xinjie Liu, Xinwei Zhang, Xianlian Gao, Weigang Wang

**Affiliations:** 1 Key Laboratory of Digital Earth Science, Aerospace Information Research Institute, Chinese Academy of Sciences, Beijing 100094, China; duss@radi.ac.cn (S.D.); liuxj@radi.ac.cn (X.L.); 2 College of Resources and Environment, University of Chinese Academy of Sciences, Beijing 100049, China; 3 Beijing Institute of Spacecraft System Engineering, Beijing 100094, China; zhangers79@163.com; 4 Academy of Inventory and Planning, National Forestry and Grassland Administration, Beijing 100714, China; gaoxianlian@afip.com.cn; 5 Beijing Institute of Space Mechanics and Electricity, China Academy of Space Technology, Beijing 100094, China; wangwg_bisme@spacechina.com

**Keywords:** solar-induced chlorophyll fluorescence (SIF), Terrestrial Ecosystem Carbon Inventory Satellite (TECIS-1), SIF imaging spectrometer (SIFIS)

## Abstract

The global monitoring of solar-induced chlorophyll fluorescence (SIF) using satellite-based observations provides a new way of monitoring the status of terrestrial vegetation photosynthesis on a global scale. Several global SIF products that make use of atmospheric satellite data have been successfully developed in recent decades. The Terrestrial Ecosystem Carbon Inventory Satellite (TECIS-1), the first Chinese terrestrial ecosystem carbon inventory satellite, which is due to be launched in 2021, will carry an imaging spectrometer specifically designed for SIF monitoring. Here, we use an extensive set of simulated data derived from the MODerate resolution atmospheric TRANsmission 5 (MODTRAN 5) and Soil Canopy Observation Photosynthesis and Energy (SCOPE) models to evaluate and optimize the specifications of the SIF Imaging Spectrometer (SIFIS) onboard TECIS for accurate SIF retrievals. The wide spectral range of 670−780 nm was recommended to obtain the SIF at both the red and far-red bands. The results illustrate that the combination of a spectral resolution (SR) of 0.1 nm and a signal-to-noise ratio (SNR) of 127 performs better than an SR of 0.3 nm and SNR of 322 or an SR of 0.5 nm and SNR of 472 nm. The resulting SIF retrievals have a root-mean-squared (RMS) diff* value of 0.15 mW m^−2^ sr^−1^ nm^−1^ at the far-red band and 0.43 mW m^−2^ sr^−1^ nm^−1^ at the red band. This compares with 0.20 and 0.26 mW m^−2^ sr^−1^ nm^−1^ at the far-red band and 0.62 and 1.30 mW m^−2^ sr^−1^ nm^−1^ at the red band for the other two configurations described above. Given an SR of 0.3 nm, the increase in the SNR can also improve the SIF retrieval at both bands. If the SNR is improved to 450, the RMS diff* will be 0.17 mW m^−2^ sr^−1^ nm^−1^ at the far-red band and 0.47 mW m^−2^ sr^−1^ nm^−1^ at the red band. Therefore, the SIFIS onboard TECIS-1 will provide another set of observations dedicated to monitoring SIF at the global scale, which will benefit investigations of terrestrial vegetation photosynthesis from space.

## 1. Introduction

Solar-induced chlorophyll fluorescence (SIF) is a re-emitted spectral signal covering the spectral range from about 650 to 850 nm with two peaks at 685 and 740 nm [1]. In recent decades, SIF has proved to be a better proxy for gross primary production (GPP) than vegetation indices (VIs) due to its direct link with plant photosynthesis [2,3,4,5,6,7,8,9,10,11]. Specifically, SIF is directly related to the actual electron transport rate, as shown by a set of coupled fundamental equations, derivation processes, and analyses of the observed SIF [12]. 

Due to the feasibility and ease of making remote sensing measurements of SIF, a flurry of research in this area has been stimulated, which has included ground-based [13,14,15,16,17,18,19], tower-based [20,21,22], airborne [17], and satellite-based [10,23,24,25,26,27,28,29,30,31,32] observations, retrieval studies, and applications. Currently, multiple satellite observations are being used for SIF retrieval at a global scale. The main satellite-based instruments used for SIF retrieval can generally be divided into two categories based on the spectral resolution. The first type is dedicated to measuring atmospheric CO_2_ and includes those on the Japanese Greenhouse gases Observing SATellite (GOSAT) [24,29,30], Greenhouse gases Observing Satellite-2 (GOSAT-2), and Orbiting Carbon Observatory-2 (OCO-2) [10,33], and also the Chinese Carbon Dioxide Observation Satellite Mission (TanSat) [32]. All of these instruments have a very high spectral resolution ranging from 0.025 to 0.044 nm. The instruments in the other category are designed to monitor distributions of various chemical trace gases in the atmosphere and include the Global Ozone Monitoring Experiment 2 (GOME-2) [25,26,28] onboard MetOp-A/B, the SCanning Imaging Absorption spectroMeter for Atmospheric CHartograhY (SCIAMACHY) [26,34] onboard ENVIronmental SATellite (ENVISAT), and the latest TROPO-spheric Monitoring Instrument (TROPOMI) [27,31] onboard Sentinel-5P. All of these instruments have a moderate spectral resolution of ~0.5 nm. All of the above satellite systems have been utilized to retrieve far-red SIF signals, whereas the GOME-2 and SCIAMACHY [26] instruments have been able to successfully detect red SIF. This was possible because of these instruments’ wider spectral coverage. 

With the rapid development of high-resolution sensors and the increasing interest in SIF research, satellite-based SIF has been widely used in quantifying GPP in recent years [4,10]. However, because of the difficulty in retrieving SIF in the red band, most research studies have only utilized the SIF from the single band around the far-red peak of the SIF emission spectrum to link with GPP. Although most studies on satellite-based SIF monitoring have focused on far-red or near-infrared (NIR) SIF retrieval due to the higher intensity of the signals and greater ease of retrieval compared to the red band, the use of red SIF signals has several advantages. First, the red-band SIF may have more potential for GPP estimation due to its link to photosystemII (PSII). The two photosystems involved in the SIF emission spectrum are photosystemI (PSI) and PSII, contributing to SIF emissions at different bands [1]. Specifically, the red SIF signals are entirely contributed to by PSII, and are directly linked to vegetation photosynthesis. The far-red SIF emissions, however, are related to both PSI and PSII. In general, the SIF quantum yield of PSII is stronger than that of PSI [1]. In addition, previous studies have shown that PSII is affected by physiological regulation, biochemical compositions, and leaf structure, whereas PSI is not affected by the biochemistry. Thus, the red SIF contains more information about PSII, and so can be used to complement the far-red SIF and make a link to GPP [35]. Secondly, the combination of red and far-red SIF information could extend the range of SIF applications. For example, the ratio of the far-red peak to the red fluorescence peak can be used as a good indicator: It is correlated with the chlorophyll content due to the stronger absorption by chlorophyll at the red band compared to that in the far-red region [36]. In addition, several studies have demonstrated that this ratio can be used to estimate the nitrogen uptake [3,37,38] and also be used in monitoring crop stress [39,40,41], due to drought and temperature, etc. [42], because of its high sensitivity to these factors. Thirdly, supplementing far-red SIF signals with red SIF can improve information about the vegetation canopy structure [1], which, inevitably, has an influence on the relationship between the canopy-level SIF and GPP. Most recently, two relevant studies that focused on the use of SIF to estimate GPP illustrated the importance of the red-band SIF to deriving GPP. One demonstrated that the combination of SIF687, SIF720, and SIF761 produced better correlations with GPP than single-band far-red or NIR SIF using continuous ground-based SIF measurements and the carbon flux dataset [43]. In the other study, it was found that the correlation between the red-band SIF at the photosystem level and the GPP is much better than that at the canopy level and also better compared to that in the NIR band using a simple reflectance-based downscaling approach [44]. This indicated that the red-band SIF has more potential for monitoring GPP if the problems of retrieval and estimation of the escape probability from the photosystem level to canopy level can be solved.

With the rapid development of remote sensing techniques, existing satellites or sensors have been successfully used to conduct SIF retrievals; however, they were originally intended for making atmospheric trace-gas measurements and may not be optimal for SIF retrievals and applications. There are no operational instruments in orbit specifically designed for SIF monitoring yet. The FLuorescence EXplorer (FLEX) will carry the FLuORescence Imaging Spectrometer (FLORIS) [45,46] as a payload. This was selected as ESA’s 8th Earth Explorer 8 mission in 2015 and is the first satellite sensor specifically designed for monitoring terrestrial vegetation SIF signals. It will be launched in 2022. 

The Terrestrial Ecosystem Carbon Monitoring Satellite (TECIS-1) is a collaborative mission approved by the China National Forestry Administration and China Academy of Space Technology. Scheduled to launch in 2021, this is the first Chinese scientific experimental satellite dedicated to the comprehensive monitoring of terrestrial ecosystems. Its aim is to monitor terrestrial ecology and resources and provide measurements that will be used to evaluate major national ecological projects. The Chlorophyll Fluorescence Hyper-spectral Monitor (SIF Imaging Spectrometer (SIFIS)) is one of the three payloads onboard TECIS-1. It is a push imaging spectrometer that will cover the spectral range from the red to NIR bands and has a spectral resolution of about 0.3 nm. Details of the characteristics of satellite instruments used for global SIF retrieval are listed in Table 1.

In this context, the SIFIS onboard TECIS-1 will be the first Chinese satellite payload specifically dedicated to measuring SIF. The key problem is to evaluate and optimize the specification of the SIFIS payload at an early stage before its launch. In this paper, we aim to evaluate SIF retrievals made with the SIFIS spectral specifications—including spectral resolution (SR), range, and signal-to-noise ratio (SNR)—and to develop an SIF retrieval algorithm for both the red and NIR bands before its launch. We also aim to investigate prospects of the SIFIS for global SIF retrieval and provide more optimal SR and SNR specifications.

## 2. Materials and Methods

### 2.1. The TECIS-1 Satellite and SIF Payload

**Figure 1:**
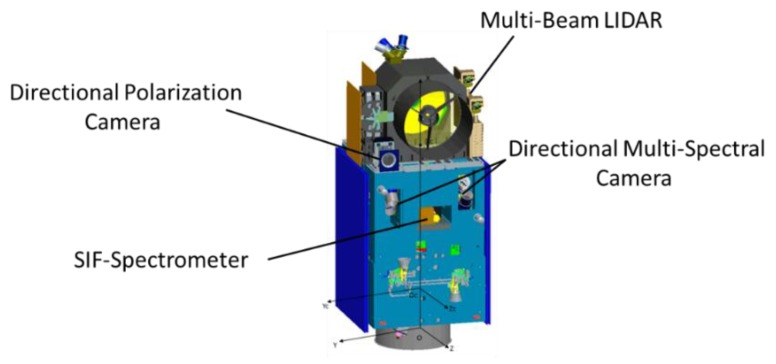
The TECIS-1 satellite layout.

The Terrestrial Ecosystem Carbon Monitoring Satellite (TECIS-1) is intended to evaluate forest biomass, measure atmospheric aerosol content, and detect photosynthetic fluorescence. These measurements will contribute to efforts to combat global warming. The satellite is designed to carry 4 payloads: Multi-Beam LIDAR, Directional Multi-Spectral Camera, Directional Polarization Camera, and Chlorophyll Fluorescence Hyper-Spectral Monitor (SIFIS), as shown in Figure 1. 

**Figure 2:**
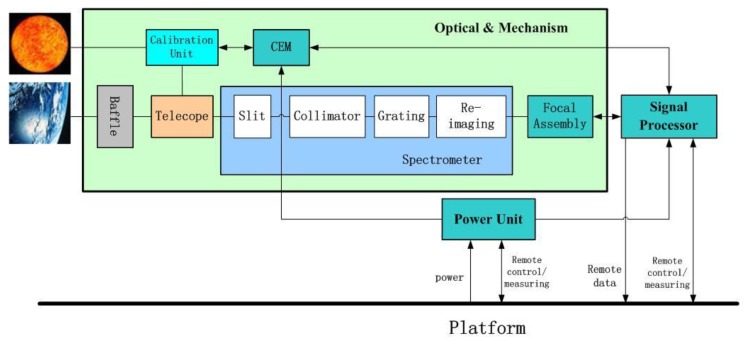
The concept of the SIF Imaging Spectrometer (SIFIS).

The satellite will operate in a sun-synchronous orbit at a height of 506 km and have a 10:30 AM local time in the descending mode. It will be launched in 2021 with a designed lifetime of 8 years. The SIFIS includes the optical mechanical main body, signal processor unit, and power unit. A calibration unit is mounted in the main body for absolute calibration in orbit, as shown in Figure 2.

**Figure 3:**
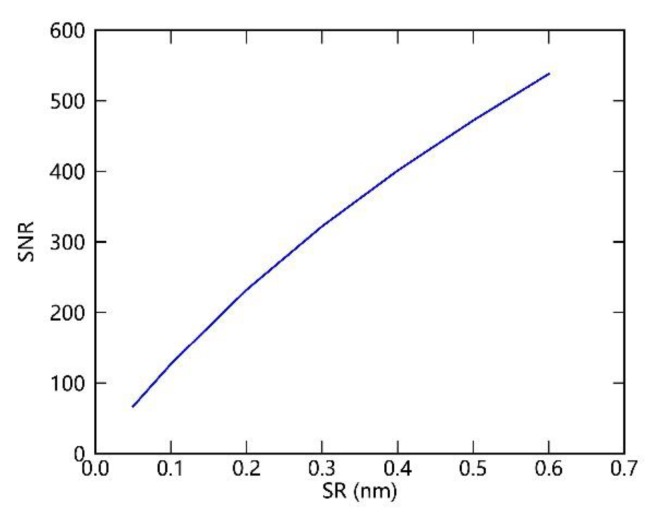
Curve of signal-to-noise ratio (SNR) versus spectral resolution (SR) provided by the instrument manufacturer.

The SIFIS aims to have a sub-nanometer SR of 0.3 nm and SNR of greater than 300. It is well known that the characteristics of the SNR and SR of a spectrometer are mutually limited by the defined binning scheme of pixels according to

(1) $$\mathrm{SNR}=\frac{S_{e}}{N_{e}}\cdot \sqrt{K},$$

where $N_{e}$ refers to the number of noise electrons, which includes the readout noise, dark noise, and shot noise. K is the number of combined pixels and $S_{e}$ is the number of electrons in the measured signal, which is related to the SR. Based on the current performance of the instrument and Equation (1), a graph of the relationship between the SR and SNR can be calculated, as shown in Figure 3. Specifically, the specification of SNR can achieve approximately 322 at a given SR of 0.3 nm. However, up to now, the specifications for the SNR under an SR of 0.3 nm are still in the optimization process. The SNR of the spectrometer can be improved through merging the imaging pixels or decreasing the electronic noise (from 0.4 to 0.2 mV), which can be obtained by increasing the power filtering or decreasing the operating temperature of the CCD (from 15 ℃ to 5 ℃). In addition, according to the information provided by the instrument manufacturer, an SNR greater than 450 can be achieved in the future. 

### 2.2. Simulated Experiment

In order to assess the retrieval characteristics under different combinations of SIFIS instrument specifications, we carried out an extensive set of simulations by combing the Soil Canopy Observation Photosynthesis and Energy (SCOPE) and Moderate resolution atmospheric TRANsmission (MODTRAN) models to generate high-spectral-resolution radiance spectra at the top of the atmosphere (TOA). 

**Figure 4:**
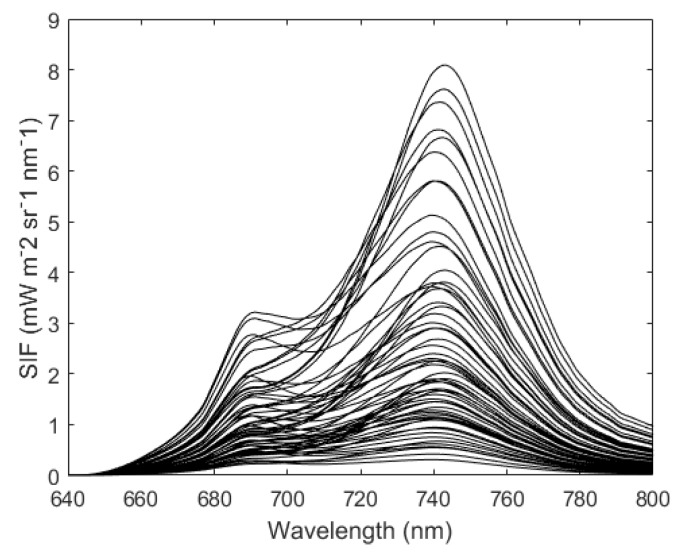
Fluorescence spectra at the top-of-canopy (TOC) level as simulated using the SCOPE model.

SCOPE [47], a vertically integrated radiative transfer and energy balance model, is widely used to simulate the canopy radiative transfer process, especially to generate the leaf and top-of-canopy (TOC) reflectance and SIF spectra. In this study, the SCOPE model (version 1.70) was employed to simulate the canopy SIF and reflectance spectra based on a look-up-table (LUT) method combining different parameters that affect the SIF simulations. The main input parameters to the SCOPE model used in this study are listed in Table 2; all other input parameters were set to their default values. This gave 60 pairs of canopy reflectance and simulated SIF values according to the combinations in Table 2, as shown in Figure 4. The spectral shape of the SIF spectra, including two peaks at the vegetation canopy level, is clearly shown in Figure 4. 

MODTRAN [48,49] is an atmospheric radiative transfer model that has a high spectral resolution of about 0.005 nm. In this study, MODTRAN 5 was used to simulate the radiation and upward atmospheric transmittance used in simulating TOA radiances in the spectral range from 600 to 800 nm. The solar irradiance data also included in this model consisted of the standard Kurucz solar data [50]. The MODTRAN 5 input parameters are listed in Table 3; there was a total of 4608 combinations. 

The radiance at the TOA received by the sensor instrument over the fluorescent vegetation surface can be represented as [51,52]

(2) $$L_{TOA}=L_{0}+\frac{\left[L_{TOC}\cdot \rho_{s}+SIF\right]\cdot T_{↑}}{1-S\cdot \rho_{s}},$$

where $L_{0}$ and $L_{TOC}$ are the atmospheric path radiance and solar radiation arriving at the surface, respectively, $\rho_{s}$ is the surface reflectance, S is the atmospheric spherical albedo, SIF is the TOC SIF signal, and $T_{↑}$ is the upward atmospheric transmittance. Similarly, the TOA radiance over non-fluorescent surfaces can be calculated by leaving out the SIF term in Equation (2).

**Figure 5:**
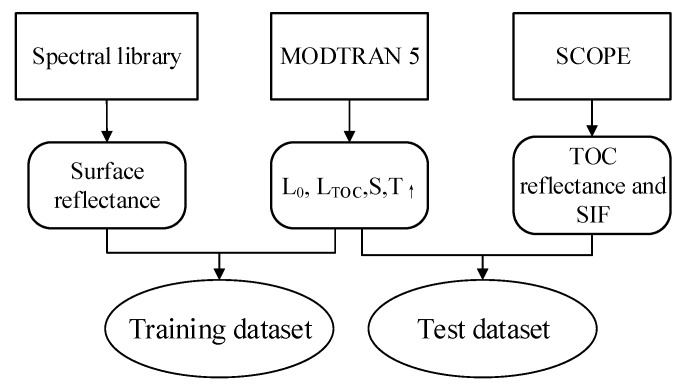
Flow-chart of the process used to produce the simulated dataset.

**Figure 6:**
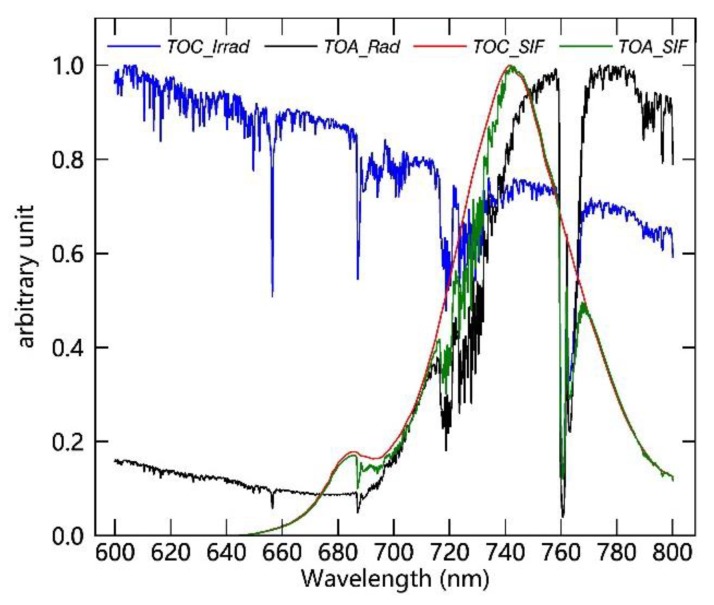
A single group of normalized simulated top-of-atmosphere (TOA) radiance spectra over vegetated surfaces (black), incident solar radiance reaching the top-of-canopy (TOC) (blue), TOC SIF (red), and TOA SIF (green) with 0.3 nm spectral resolution and 0.1 nm sampling interval derived from SCOPE and MODTRAN 5 models.

In this paper, the principal component analysis (PCA) [53] data-driven SIF retrieval method was used to evaluate the SIF retrieval performance using the simulated dataset. For this data-driven method, SIF is regarded as an additive signal; thus, the TOA radiance can be represented as the sum of the fluorescent component and non-fluorescent component, as shown in Equation (2). Detailed descriptions for this method can be found in Section 2.3. Thus, in order to make use of this method, two separate datasets are needed simultaneously. One of these, denoted as the training dataset, should not include the SIF contributions used for deriving the principal components; and the other, denoted as the test dataset, should contain the SIF signals used to assess the retrieval results under different settings. A schematic of the process for producing the simulated datasets is shown in Figure 5. First, three groups of TOA radiance spectra can be obtained, thus giving three reflectance values that can be used as input parameters for the MODTRAN model. Next, the relevant atmospheric radiation transfer parameters ($L_{0}$, $L_{TOC}$, and S) used in simulating the training and test datasets can be calculated using an algebraic method based on Equation (2). Seven different non-fluorescent surface reflectance spectra corresponding to snow and bare soil surfaces, derived from the ENVI (Environment for Visualizing Images) spectral library [54], were used to derive the training dataset. Then, 60 pairs of canopy reflectance and SIF spectra derived using the SCOPE model were used to derive the test dataset. In addition, the training and test TOA radiance spectra with an original spectral resolution of 0.005 nm were calculated by combining the surface reflectance or TOC SIF and reflectance with the atmospheric parameters according to Equation (2). Eventually, a total of 32,256 training samples and 276,480 test samples were produced, respectively. Then, the high-spectral-resolution TOA spectra were convolved to lower-resolution spectra using a Gaussian filter with a full-width at half-maximum (FWHM) of 0.3 nm. An example of a group of simulated spectra with an SR of 0.3 nm is displayed in Figure 6: This includes spectra of TOA radiance over vegetated surfaces, incident radiance reaching the vegetation top-of-canopy (TOC), TOA SIF, and TOC SIF. Both the oxygen and solar absorption features can be clearly seen in Figure 6. 

In this paper, except for the simulated spectra dataset with an SR of 0.3 nm and SNR of 322, pre-configured for the SIFIS currently, similar simulation experiments with different combinations of SR and SNR are also needed to investigate the prospects of these configurations for SIF retrieval. In addition, we assumed that the noise followed a Gaussian distribution and was independent of radiance level. Thus, according to different evaluating purposes, four different simulation experiments were conducted based on the above training and test simulated datasets, which have an original spectral resolution of 0.005 nm, as shown in Table 4. First, in order to investigate the performance of SIF retrieval using simulated TOA spectra based on the current instrument level, as shown in Figure 3, one experiment denoted as Exp I in Table 4 was conducted to simulate three groups of simulations, including simulated spectra combining an SR of 0.3 nm and SNR of 322, an SR of 0.1 nm and SNR of 127, and an SR of 0.5 nm and SNR of 472. Secondly, one experiment that contains five groups of simulations, denoted as Exp II in Table 4, was conducted to investigate the effects of SR on the SIF retrievals under two given SNR cases, noise-free and SNR of 322. Thirdly, as an SNR of 450 at a given SR of 0.3 nm will be potentially achieved, one simulated dataset with this combination was included in Exp III, which was employed to investigate the SIF retrieval performance under this optimized specification in the future. Lastly, in order to investigate the effects of the SNR on the retrieval precision at given SRs, Exp IV included two different datasets with different noise added to simulations for a given SR of 0.1 and 0.3 nm. Detailed information of these simulation experiments is shown in Table 4.

### 2.3. Data-Driven SIF Retrieval Method

Similar to Equation (2), assuming Lambertian reflectance over the fluorescent surface, the radiance at the TOA received by the sensor can also be represented as a superposition of the radiation reflected by the vegetation surface and the SIF signals [31], displayed as follows:

(3) $$L_{TOA}=\frac{I_{sol}\cdot \mu_{0}}{\pi }\left(\rho_{0}+\frac{\rho_{s}\cdot T_{↓↑}}{1-S\cdot \rho_{s}}\right)+\frac{F_{s}\cdot T_{↑}}{1-S\cdot \rho_{s}},$$

where $I_{sol}$ is the solar irradiance at the TOA level, $\mu_{0}$ is the cosine of the solar zenith angle, $\rho_{0}$ is the atmospheric path reflectance, and $T_{↓↑}$ is the two-way atmospheric transmittance, i.e., the product of the downward and upward atmospheric transmittance. The main challenge is to separate the SIF from the radiance reflected by the surface, which is more than 100 times the intensity of the SIF signal [28]. As stated in previous work, the spectrally smooth components, including contributions from the surface reflectance ($\rho_{s}$) and the atmospheric scattering effects ($\rho_{0}$ and S), can be modeled using a low-order polynomial, which is a function of the wavelength; the spectrally oscillating component, which is mainly due to the atmospheric absorption effect ($T_{↓}$ and $T_{↑}$), can be reconstructed using a statistical approach [25,26,31]. Principal component analysis (PCA) is a statistical procedure that uses an orthogonal transformation to convert a set of observations of possibly correlated variables into a set of values of linearly uncorrelated variables called principal components, and is widely used in the decomposition and reconstruction of signals [14,15]. In recent years, it has been used to replace the full-physics method to model the atmospheric absorption characteristics for satellite SIF retrievals [25,26,31].

Assuming that the SIF is Gaussian-like within the fitting window, a normalized and fixed spectral function $h_{f}$ can be formulated:

(4) $$h_{f}=\exp\left[\frac{-{\left(\lambda -\lambda_{0}\right)}^{2}}{2\sigma_{h}{}^{2}}\right],$$

where $\lambda_{0}$ is the wavelength of the SIF peak emission within the fitting window and the value of $\sigma_{h}$ depends on the spectral fitting window selected for SIF retrieval. In order to simplify the non-linear problem, the effective upward transmittance, $T_{↑}^{e}$ [25], shown in Equation (5), is also used to replace $T_{↑}$ in the forward model.

(5) $$T_{↑}^{e}=\exp\left[\ln\left(T_{↓↑}^{e}\cdot \frac{\sec\left(\theta_{v}\right)}{\sec\left(\theta_{v}\right)+\sec\left(\theta_{o}\right)}\right)\right],$$

where $T_{↓↑}^{e}$ is the effective two-way atmospheric transmittance derived from the training dataset by normalizing the solar irradiance radiance spectra with respect to the low-order polynomials, $\theta_{o}$ denotes the solar zenith angle (SZA), and $\theta_{v}$ is the viewing zenith angle (VZA). Although there is a difference between $T_{↑}^{e}$ and $T_{↑}$ over vegetated surfaces because of the in-filling of the SIF, this difference is negligible according to the results of the assessment conducted by Köhler, Guanter, and Joiner [28]. Thus, the final forward model derived from Equation (3) can be simplified to

(6) $$L_{TOA}\left(\alpha ,\beta ,F_{s}\right)=I_{sol}\cdot \frac{\mu_{0}}{\pi }\cdot \overset{n_{p}}{\underset{i=0}{\sum }}\left(\alpha_{i}\cdot \lambda^{i}\right)\cdot \overset{n_{pc}}{\underset{j=1}{\sum }}\left(\beta_{j}\cdot PC_{j}\right)+F_{s}\cdot h_{f}\cdot T_{↑}^{e},$$

where $\alpha_{i}$ and $\beta_{j}$ are coefficients of the polynomial and the principal components, respectively, $F_{s}$ is the SIF at the required wavelength, λ is the wavelength, $n_{p}$ is the order of polynomial, and $n_{pc}$ is the number of principal components selected. Based on this simplified forward model (Equation (6)) and the selected PCs, two different fitted TOA spectra can be calculated to demonstrate the suitability of the PCA method. One includes the SIF signal during the fitting process and the other does not take SIF into account.

**Figure 7:**
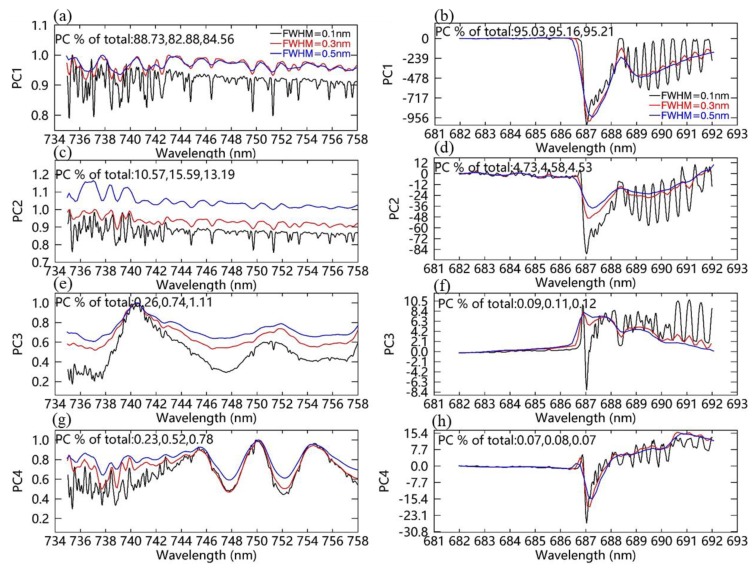
The leading four principal components (PCs) of noise-free simulated spectra for different SRs of 0.1 nm (black), 0.3 nm (red), and 0.5 nm (blue). (**a**,**c**,**e**,**g**) are the first leading PCs for the far-red band, and (**b**,**d**,**f**,**h**) are the first leading PCs for the red band. The explained variances of each PC for three SRs are also listed at the top of each graph.

In this study, we chose two retrieval fitting windows ranging from 735 to 758 nm and 682 to 692 nm to retrieve the SIF signals at the far-red and red band, respectively. Depending on the width and complexity of the fitting windows, we normalize the spectra with respect to 2-order and 3-order polynomial fits at wavelengths that are not significantly affected by atmospheric absorption. As for the reference SIF spectral shape, $\sigma_{h}$ was set to 21 and 9.5 for the far-red and red bands, respectively. The selection of the threshold used in this study was based on the experience acquired in previous studies [30] and was intended to be a compromise between dealing with the overfitting problem and guaranteeing the goodness of fit at the same time. Compared to the red band fitting window, the far-red band window is less complicated as it is mostly affected by the weak water absorption without any atmospheric absorption. The red band fitting window is affected by oxygen absorption, and there is a much deeper absorption depth in the O_2_-B band (centered at about 687 nm), as shown in Figure 7 and Figure 12. 

### 2.4. Metrics Used for Accuracy Assessment

In this paper, the simulated SIF spectra over the red and far-red fitting windows for 60 simulated vegetation canopy conditions are averaged and selected as true red and far-red band SIF values. In order to evaluate the performance of the SIF retrievals using simulated spectra with different combinations of SR and SNR, statistical parameters were used. Here, the commonly used parameters including the root-mean-squared difference (RMS diff), correlation coefficient (r), standard deviation (σ), bias between the retrieved and true SIF, and the slope and intercept of the linear fit were calculated and averaged for all atmospheric and geometrical conditions (a total of 4608 scenarios). In particular, systematic errors will inevitably be produced using the data-based retrieval method. Thus, in order to separate the influence of systematic effects due to the adopted method from those due to the different specifications, the corrected RMS difference (RMS diff*), which was based on comparisons between the true SIF values and the corrected SIF retrievals ($SIF_{corr}$) without any systematic effects, was also calculated. Making use of the statistical parameters for the linear fit between the true SIF and retrieved SIF ($SIF_{retrieved}$), the corrected retrieved SIF values were calculated using

(7) $$SIF_{corr}=\frac{SIF_{retrieved}-bias}{slope}.$$

## 3. Results

### 3.1. Performance of the PCA Data-Driven Approach for Fitting the TOA Radiance

Based on the cumulative variance explained by the principal components (PCs) derived from the simulated spectra of non-vegetated surfaces, the number of PCs used to reconstruct the SIF-free spectrum was determined by a threshold of 99.95% [32]. In the end, 8 far-red PCs and 10 red PCs were selected in the retrieval process. Figure 7a,b show the four leading PCs for the far-red and red fitting windows, respectively, which were computed using noise-free simulated data with SRs of 0.1, 0.3, and 0.5 nm. The explained variances for each PC of three simulations with SRs of 0.1, 0.3, and 0.5 nm are also displayed in each subplot, and they vary slightly for different SRs. The spectral variance in the far-red fitting window is mainly due to absorption by water vapor, as shown in Figure 7a, while it is due to the larger O_2_-B absorption band, as well as the Fraunhofer lines, in the red window, as shown in Figure 7b. For each fitting window, the spectral shapes of the PCs for the data simulated with the three different SRs are similar. The obvious difference is that much more detailed spectral information can be obtained by the simulated data with the higher SR of 0.1 nm. However, it should be noted that the first PC can capture almost all of the spectral variance for all of the simulations using the three different SRs. 

**Figure 8:**
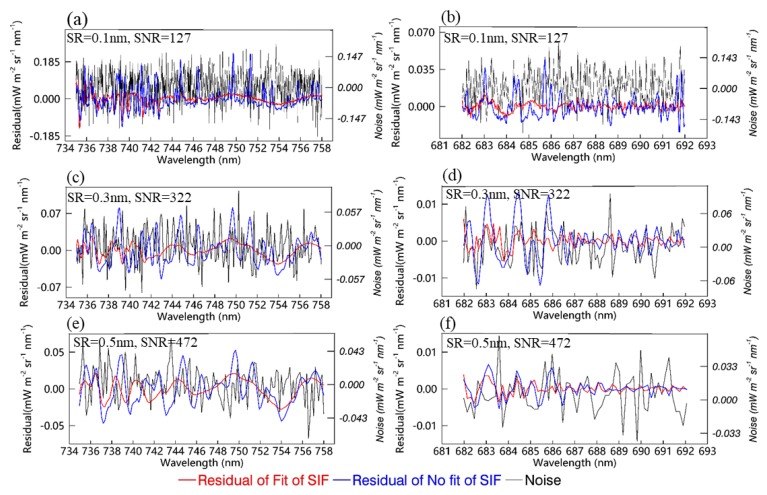
The spectral residuals between the fitted and measured TOA radiances when the retrieved SIF is fitted (blue) and not fitted (red), as well as the noise spectrum (black). (**a**,**c**,**e**) are the spectral residuals for the far-red band with an SR of 0.1 nm and SNR of 127, an SR of 0.3 nm and SNR of 322, and an SR of 0.5 nm and SNR of 472, respectively; (**b**,**d**,**f**) are those for the red band, respectively.

Two different fitted TOA radiance spectra, SIF contributions included and not included, were obtained by making use of the simplified forward model and the selected PCs. Then, residual spectra between the measured TOA radiance and fitted radiance were averaged for a total of 276,480 simulated TOA radiances. Figure 8a,c,e show the averaged spectral residuals over the far-red band, and Figure 8b,d,f show those over the red band. It can be seen that the spectral residuals are much smaller when the SIF is included, particularly at the absorption lines. In addition, the spectral residuals for the case with low SR and high SNR (as shown in Figure 8a,b) are smaller than for other cases at the red and far-red windows, which indicates that the magnitude of the residuals is primarily dependent on the SR. Further comparisons between the spectral residuals for different SRs showed that the range of the averaged residual is larger for lower SRs, and the spectral residual for an SR of 0.1 nm and SNR of 127 (Figure 8a,b) is about 1.59 times that for an SR of 0.3 nm and SNR of 322 (Figure 8c,d). However, compared to the absolute radiance (usually greater than 100 mW m^−2^ sr^−1^ nm^−1^) and SIF (greater than 1 mW m^−2^ sr^−1^ nm^−1^ for a dense canopy and the simulated SIF magnitude shown in Figure 4), the magnitude of the residuals is almost negligible (the average absolute value is <0.02 and <0.003 mW m^−2^ sr^−1^ nm^−1^ at far-red and red band, respectively). The spectral residuals for different SRs vary from −0.15 to 0.10 mW m^−2^ sr^−1^ nm^−1^ at the far-red fitting window, and from −0.01 to 0.01 mW m^−2^ sr^−1^ nm^−1^ at the red fitting window. The above results show that the PCA method can be successfully used to reconstruct the shape of the spectra for SIF-free surfaces, which also implies that SIF signals can be discriminated from satellite-based TOA radiance using this data-driven method.

### 3.2. Performance of the PCA Data-Driven Approach for SIF Retrieval

**Figure 9:**
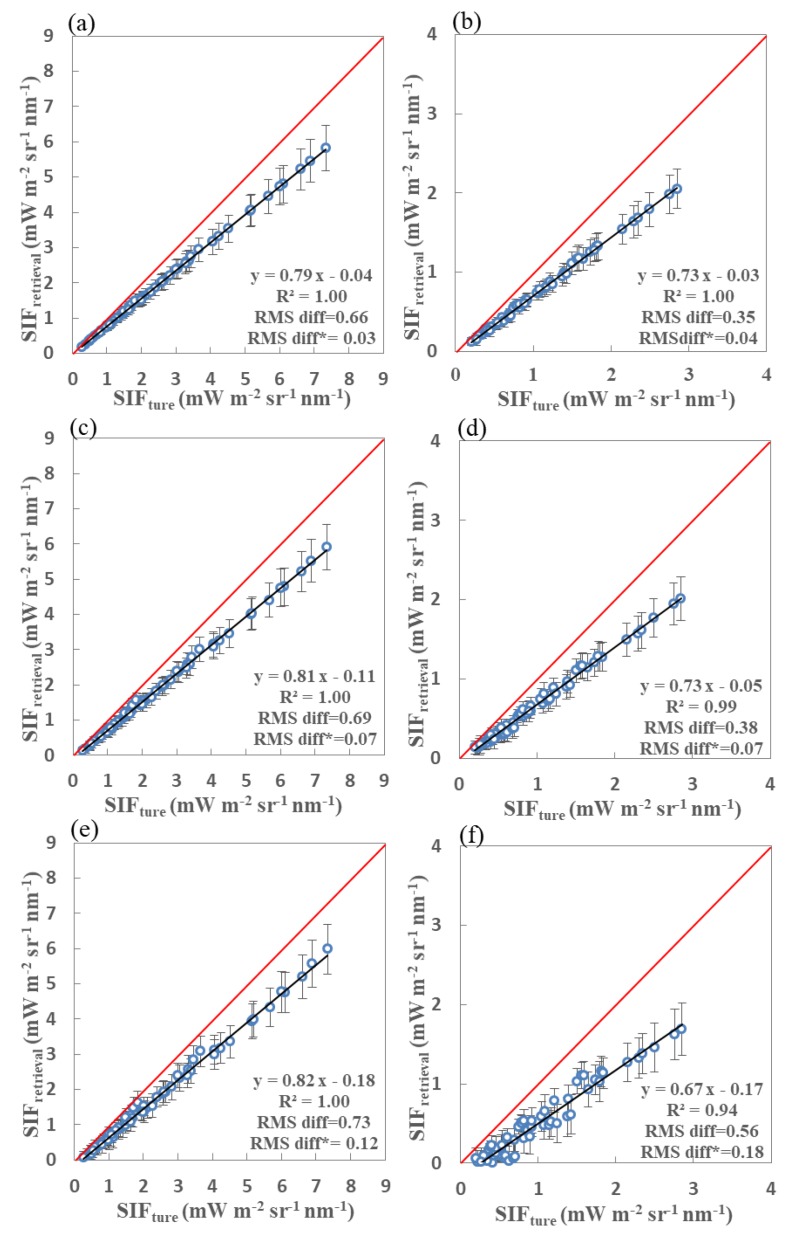
Retrieved vs. true fluorescence using far-red (**a**,**c**,**e**) and red (**b**,**d**,**f**) fitting windows derived from noise-free simulated data with spectral resolutions of 0.1 nm (**a**,**b**), 0.3 nm (**c**,**d**), and 0.5 nm (**e**,**f**). Standard derivation error bars are also shown as vertical lines for each vegetation canopy type. The true SIF values at each band are averaged over the fitting windows for 60 simulated vegetation canopy conditions. The circles mark the averaged retrieved SIF value for a total of 4608 atmospheric and geometrical conditions.

In order to display the performance of the SIF retrieval using the simulated spectra covering both the far-red and red fitting windows, the retrieved SIF values were plotted against the true values at both the far-red and red bands for two sets of noise-free simulations with SRs of 0.1, 0.3, and 0.5 nm. The results of this are shown in Figure 9. From Figure 9a,c,e, it can be concluded that the retrieval accuracy and precision obtained using the noise-free simulated data with SRs of 0.1, 0.3, and 0.5 nm at the far-red band decrease slightly with low SRs, with RMS diff* values of 0.03, 0.07, and 0.12 mW m^−2^ s^−1^ nm^−1^, respectively. In addition, the differences between the results obtained using three different SRs are negligible in terms of the statistical parameters RMS diff and RMS diff*. As for the red band, Figure 9f shows that the retrieved SIF values for the SRs of 0.5 nm are much worse than those for the simulations with SRs of 0.1 and 0.3 nm, as shown in Figure 9b,d, respectively; however, the retrieval errors are still all less than 20%. Thus, both far-red and red SIF signals can be successfully obtained using satellite-based observations covering both the above spectral ranges if the SNR is adequate. 

### 3.3. Performance of SIF Retrievals Using Simulations with Different SRs and SNRs

**Figure 10:**
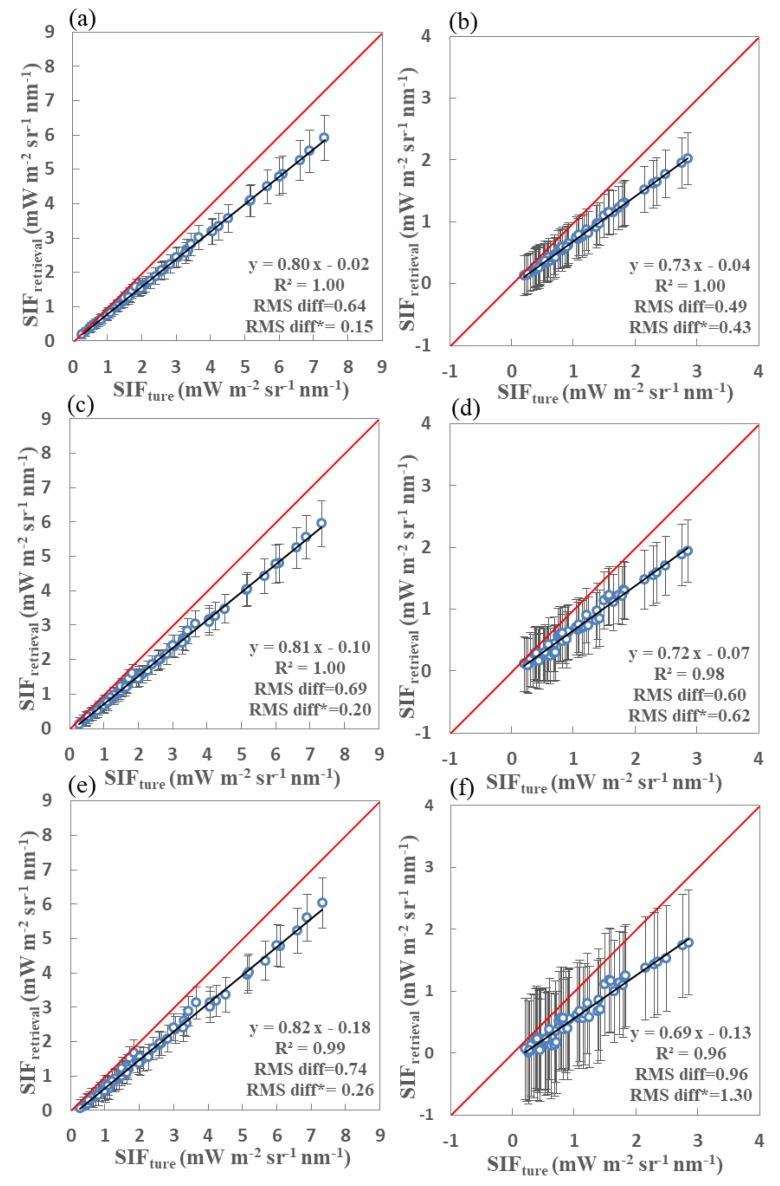
Retrieved vs. true fluorescence using far-red (**a**,**c**,**e**) and red (**b**,**d**,**f**) fitting windows derived from simulated data with an SNR of 127 and SR of 0.1 nm (**a**,**b**), SNR of 322 and SR of 0.3 nm (**c**,**d**), and SNR of 472 and SR of 0.5 nm (**e**,**f**). Standard deviations are also shown as vertical bars.

For comparison, scatter plots of retrieved versus true SIF values for the far-red and red bands for three different SNR and SR combinations are displayed in Figure 10. Similar to the comparisons in Figure 9 (free of noise), Figure 10a–f represent the comparison results for simulations with SRs of 0.1, 0.3, and 0.5 nm at both far-red and red bands, in which the corresponding noise was included. The RMS diff* for the far-red SIF retrievals using simulations with an SR of 0.1 nm and SNR of 127 (0.15 mW m^−2^ s^−1^ nm^−1^) is slightly less than for the simulations with an SR of 0.3 nm and SNR of 322 (0.20 mW m^−2^ s^−1^ nm^−1^) and SR of 0.5 nm and SNR of 472 (0.26 mW m^−2^ s^−1^ nm^−1^). However, as for the red band in Figure 10b,d,f, the retrieval accuracy noticeably decreases with the decrease in SR, and the retrieval of SIF with an RMS diff* of 1.30 mW m^−2^ s^−1^ nm^−1^ is too poor for the configuration with an SR of 0.5 nm and an SNR of 472. The value of RMS diff* for the SIF results is also much higher (1.30 mW m^−2^ s^−1^ nm^−1^) for an SR of 0.5 nm and SNR of 472 than for an SR of 0.3 nm and SNR of 322 (0.62 mW m^−2^ s^−1^ nm^−1^) and an SR of 0.1 nm and SNR of 127 (0.43 mW m^−2^ s^−1^ nm^−1^). It can also clearly be seen that the biases in the large SIF values are larger for the simulations with coarser SRs.

In order to determine the best choice of SNR and SR, we conducted a further set of experiments using simulated datasets with different combinations of SNR and SR. The SIF values at both the far-red and red bands were again obtained using the fitting windows and retrieval method described above. Table 5 lists comprehensive results of the true and retrieved SIF values at both the far-red (line 1 to 9) and red bands (line 10 to 18). Statistical results for the comparisons using the simulations with different combinations of SR and SNR are also listed in Table 5. Lines 4, 5, and 6 show retrieval statistics for the far-red band based on three simulations: An SR of 0.1 nm and SNR of 127, an SR of 0.3 nm and SNR of 322, and an SR of 0.5 nm and SNR of 472. For the second case, the retrieval accuracy is rather low at the red band—the corrected RMS diff* is 0.62 mW m^−2^ s^−1^ nm^−1^. 

It is inevitable that the retrieval precision (R^2^) and accuracy (RMS diff and RMS diff*) will both increase for a higher SR and SNR. For the experiments without any noise, there is no obvious difference in retrieval performance between an SR of 0.1 and 0.3 nm at both the far-red and red bands, and both perform well. However, if the noise is included, the performance for an SR of 0.1 nm is noticeably better than for SRs of 0.3 and 0.5 nm at both bands. At the red band, in particular, there is a clear decrease in RMS diff and corrected RMS diff* for an increased SR. These improvements may be contributed to the deeper absorption lines and larger number of spectral samples used in the same fitting window when the SR is higher. However, the performance for the simulations with an SR of 0.3 nm is most likely to improve along with the simulations with an SR of 0.1 nm and SNR of 127 if the SNR is greater than 450. 

**Figure 11:**
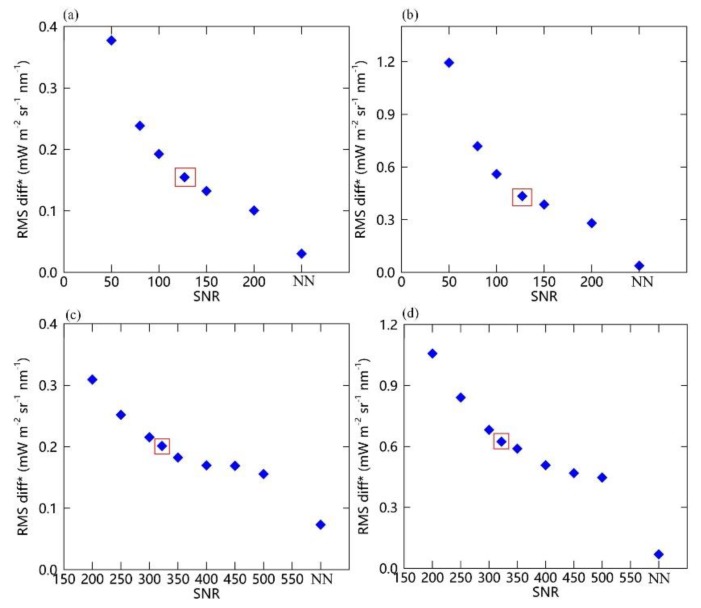
Variations of RMS diff* of SIF retrievals at far-red (**a**) and red (**b**) bands using simulations with a different SNR and SR of 0.1 nm (**a**,**b**) and 0.3 nm (**c**,**d**). ‘NN’ indicates the noise-free simulations condition. The red box refers to the RMS value of the simulations with SNRs of 127 (**a**,**b**) and 322 (**c**,**d**), respectively.

In order to clearly display the effects of the SNR on the retrieval precision, Figure 11 shows the variations in RMS diff* for the true and retrieved SIF values for different SNRs using the simulated data with SRs of 0.1 and 0.3 nm. The RMS diff* was calculated using the SIF retrievals after correction for the systematic retrieval errors. Thus, RMS diff* indicates the performance of simulated data for different SNRs at a given SR. It can clearly be seen that, at both far-red and red bands, the RMS diff* errors decrease significantly as the SNR increases for the simulations with SRs of 0.1 and 0.3 nm, which is consistent with the behavior displayed in previous studies [25,26,32]. The RMS diff* errors are clearly higher in the red band than in the far-red band. Thus, although the continuing increase in the SNR will lead to an improvement in SIF retrieval performance, according to the current specifications of the instrument, the RMS diff* based on simulations with an SR of 0.3 nm and SNR of 322 is larger than for simulations with an SR of 0.1 nm and SNR of 127, especially in the red band. This indicates that an SR of 0.1 nm is more likely to guarantee good SIF retrieval performance. However, if the SNR is improved to greater than 450, an SR of 0.3 nm will guarantee an RMS diff* almost as small as for an SR of 0.1 nm and SNR of 127.

## 4. Discussion

### 4.1. Uncertainty in the SIF Retrieval Method

**Figure 12:**
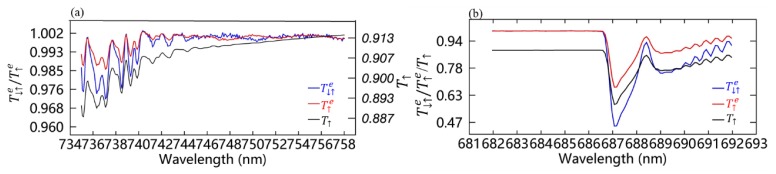
Example of the estimated effective two-way atmospheric transmittance ($T_{↓↑}^{e}$, shown in blue) and the effective upward transmittance ($T_{↑}^{e}$, shown in red) plotted against the true upward transmittance ($T_{↑}$, shown in black) at far-red band (**a**) and red band (**b**) based on simulations with an SR of 0.3 nm and SNR of 322.

As shown in Figure 9 and Figure 10, there is obvious systematic bias (about 18%–21% for far-red band and 27%–33% for red band) between the retrieved SIF and true SIF, which is inevitably produced by the data-driven retrieval algorithm. This bias can be contributed to two factors, the calculation of effective upward transmittance and the fitting bias of SIF spectral shape. The first factor is related to the difference between the true upward transmittance ($T_{↑}$) and the effective upward transmittance, $T_{↑}^{e}$, calculated based on Equation (5). $T_{↑}^{e}$ is estimated from the effective two-way atmospheric transmittance ($T_{↓↑}^{e}$), which is calculated using the apparent reflectance normalized by the low-order polynomials. In Figure 12, a schematic example of a comparison between the estimated $T_{↓↑}^{e}$ and $T_{↑}^{e}$ and the real $T_{↑}$ at both far-red and red bands is provided. It can be seen that $T_{↑}^{e}$ is higher than $T_{↑}$ across the whole of the fitting windows. This is generally because the polynomial fit of the apparent reflectance assumes that the transmittance of the fitting window is approximately 1; this is quite different from the real situation, especially for the cases of high atmospheric optical thickness or larger solar or viewing angles. The ratio of $T_{↑}^{e}$ to $T_{↑}$ is about 1.10 at the far-red band and about 1.13 at the red band. Therefore, the systemic underestimation of the retrieved SIF can be partly corrected using the ratio of $T_{↑}^{e}/T_{↑}$ determined from the simulated dataset.

**Figure 13:**
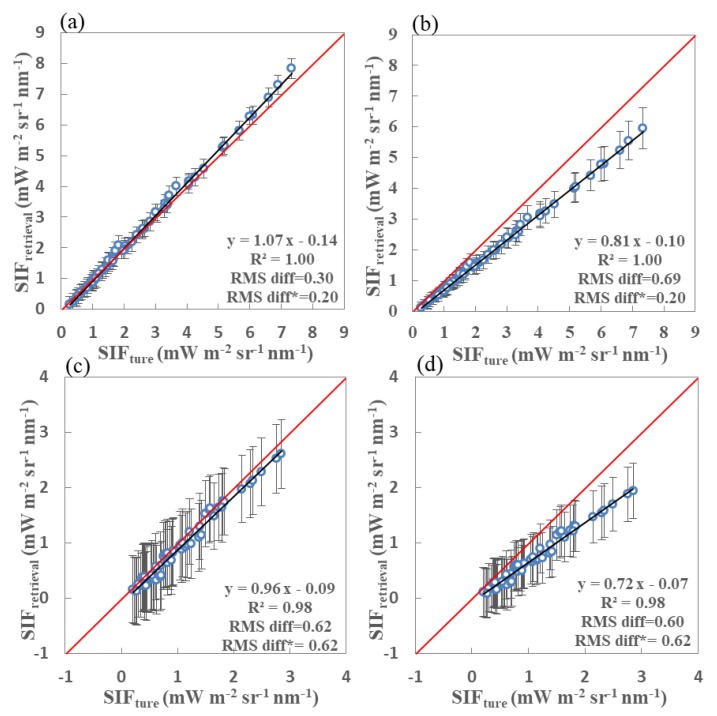
Comparisons between SIF retrievals using the true upward transmittance (**a**,**c**) and that using the effective upward transmittance (**b**,**d**) calculated by Equation (3) at both far-red (**upper panels**) and red bands (**bottom panels**). Similar to Figure 12, simulations with SR of 0.3 nm and SNR 322 are used.

In order to quantitatively investigate the effects of the use of the effective upward transmittance to retrieve the SIF, an example showing a comparison between the SIF retrieved using the true upward transmittance derived from the simulated dataset and the effective upward transmittance calculated using Equation (3) is shown in Figure 13. It is clear that, for both the far-red and red bands, the SIF points retrieved using the true upward transmittance are much closer to the 1:1 line than those obtained using the effective upward transmittance. In addition, the results retrieved using the simulations with an SR 0.3 nm and SNR of 322 show that the use of the effective upward transmittance results in an underestimation of approximately 23% and 21% at the far-red and red bands, respectively. Furthermore, as $T_{↑}^{e}$ is estimated from the training dataset and is directly applied when retrieving SIF signals using the test data, $T_{↑}^{e}$ will also slightly differ from the real $T_{↑}$ over vegetation surfaces, especially at the atmospheric absorption lines. This is due to the in-filling effect of SIF, which has been regarded as negligible in previous studies [25]. Thus, for both of these reasons, the estimation value of $T_{↑}^{e}$ can be higher than the real $T_{↑}$, which, in turn, leads to an underestimation of the SIF. 

**Figure 14:**
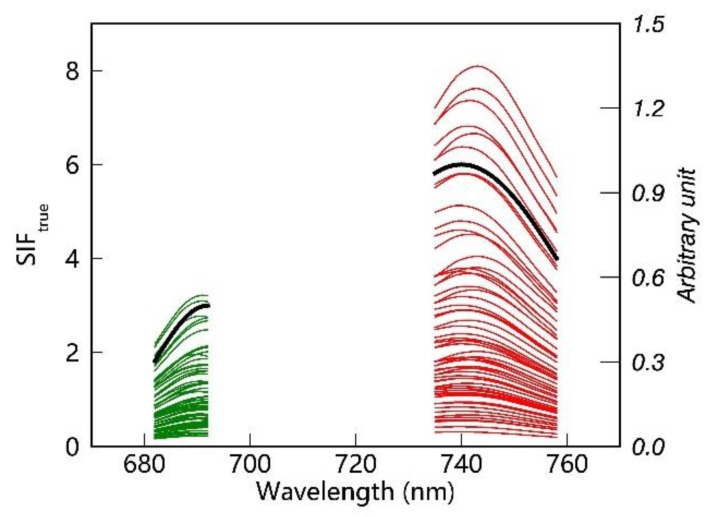
True SIF spectrum at red (green) and far-red (red) fitting windows derived using the SCOPE model and the fixed spectral function (black) based on the Gaussian function. $\sigma_{h}$ was set as 21 and 9.5 and $\lambda_{0}$ was set as 740 and 692 nm for the far-red and red band, respectively.

There are still other factors to be considered when trying to reduce the uncertainty in the SIF retrieval. For example, though the Gaussian function is usually used to model the shape of the SIF spectrum, differences between the simulated shape and the real shape within the fitting windows, which are displayed in Figure 14, will produce large uncertainties in the retrieved values. The SIF emission peaks are also variable and depend on variations in the canopy structure and leaf biochemistry, especially in the case of the red SIF emission peak [55]. A wider fitting window increases the size of the differences between the Gaussian function and the real spectrum. Specifically, different combinations of the input parameters $\sigma_{h}$ and $\lambda_{0}$ will result in big differences in the retrieved SIF values [56]. Comparisons between the simulated SIF spectra and the SIF spectra fitted using the Gaussian function and the combinations used in this paper show that the differences are about 2% and 4% at the far-red and red bands, respectively. Here, we selected the optimal combinations of $\sigma_{h}$ and $\lambda_{0}$ to conduct the retrieval process. These combinations were based on the correlation between the simulated and true SIF spectra. In addition, the accuracy and precision of the retrieved values are also highly sensitive to the PCs and fitting windows that are selected. However, the sensitivity to these factors is not our focus of this paper, and the selections used were based on the retrieval results obtained during this study.

### 4.2. Determination of SR, SNR, Spectral Range, and Other Requirements for the SIFIS and the TECIS-1 Satellite 

Through the statistical analysis of comparisons between the true and retrieved SIF values, it can be concluded that both the far-red and red SIF can be obtained successfully if the SIFIS is able to obtain measurements that cover the spectral range from the red to far-red band. In addition, due to the significant influence of water vapor and the lower SIF signals in the wavelength range from about 780 to 850 nm [46], we suggest that the spectral range for the SIFIS should be from 670 to 780 nm, which also covers the O_2_-A and O_2_-B bands. In addition, according to the above analysis, of the three simulations, the best is the combination of an SR of 0.1 nm and SNR of 127. Thus, based on the current specifications with an SR of 0.1 nm and SNR of 127 and SR of 0.3 nm and SNR of 322, the SR for the SIFIS should be set to 0.1 nm and the corresponding SNR should be greater than 127. However, the RMS diff* values of SIF retrievals at both the far-red (0.17 mW m^−2^ s^−1^ nm^−1^) and red (0.47 mW m^−2^ s^−1^ nm^−1^) bands using simulations with an SR of 0.3 nm and SNR of 450 are similar compared to using simulations with an SR of 0.1 nm and SNR of 127, with an RMS diff* value of 0.15 mW m^−2^ s^−1^ nm^−1^ at the far-red band and 0.43 mW m^−2^ s^−1^ nm^−1^ at the red band, as displayed in Table 5. As stated in Section 2.1, an SNR of 450 for an SR of 0.3 nm is likely to be achieved in the future. In addition, as suggested by the instrument manufacturer, it is easier to improve the SNR based on an SR of 0.3 nm than to improve the SR from 0.3 to 0.1 nm. Thus, if the SNR could be improved to 450, the retrieval performance for simulations with an SR of 0.3 nm would be as good as those with an SR of 0.1 nm and SNR of 127. 

In terms of SIF retrieval, as well as the requirements for the spectrometer onboard TECIS-1, several other requirements should be considered, including temporal, spatial, and local time requirements. The SIF is an instantaneous signal and will have a highly dynamic variance depending on the illumination and effects of stress and environmental conditions. A high frequency of measurements and large coverage are needed to track the dynamic variations in SIF signals. In addition, a high spatial resolution is also needed in order to investigate the photosynthetic mechanisms in vegetation on a small land scale, especially so that validation with ground-based measurements can be carried out. However, due to production constraints, these requirements cannot be satisfied simultaneously. Considering the need for a high spatial resolution and global coverage, a spatial resolution of greater than 1 km is optimal for identifying vegetation canopy types at site scales. As for the global coverage, coverage over all land surfaces and main islands should be available [46]. The SIFIS gives only a coarse resolution of 2 km and a narrow swath of 30 km, which does not match well with the requirements for the global mapping of SIF.

## 5. Conclusions

TECIS-1 is the first Chinese scientific satellite designed for the comprehensive monitoring of terrestrial ecosystems. The SIFIS onboard TECIS-1 is also the first Chinese instrument specifically designed for SIF monitoring from space. In this paper, a wide spectral window ranging from 670 to 780 nm is recommended, which will enable SIF retrievals at both the far-red and red bands. In general, there is a trade-off between spectral resolution and SNR as well as the spatial–temporal resolution. In order to guarantee SIF retrieval accuracy and precision at both the far-red and red bands, it is concluded that the optimal combination is an SR of 0.1 nm and SNR of 127. With these specifications, SIF retrievals with RMS diff* values of 0.15 and 0.43 mW m^−2^ sr^−1^ nm^−1^ for the far-red and red band, respectively, can be obtained using the PCA retrieval algorithm. However, it is very hard to improve the SR from 0.3 to 0.1 nm. For an SR of 0.3 nm, RMS diff* can be improved to 0.17 mW m^−2^ sr^−1^ nm^−1^ at the far-red band and 0.47 mW m^−2^ sr^−1^ nm^−1^ at the red band if the SNR can also be improved to 450. In conclusion, the SIFIS onboard TECIS-1 will be the first Chinese instrument specifically designed for terrestrial vegetation SIF monitoring on a global scale. 

## Figures and Tables

**Table 1:** Technical characteristics of current satellites/instruments with the ability to provide global solar-induced chlorophyll fluorescence (SIF) retrievals as well as the planned Terrestrial Ecosystem Carbon Inventory Satellite (TECIS-1) and FLuorescence EXplorer (FLEX).

Satellite/ Sensor	Data Available From	Equatorial Crossing Time	Spectral Coverage (nm)	Spectral Resolution (nm)	Spatial Resolution	Swath (km)
GOSAT	04/2009	1:00 pm	757–775	0.025	10 km diam.	750
OCO-2	08/2014	1:30 pm	757–771	0.042	1.3 km × 2.25 km	10.3
TanSat	02/2017	1:30 pm	758–778	0.044	2 km × 2 km	20
SCIAMACHY	03/2002	10:00 am	650–790	0.5	30 km × 240 km or 30 km × 60 km **	240
GOME-2	01/2007	9:30 am	650–790	0.5	40 km × 80 km or 40 km × 40 km *	1920
TROPOMI	11/2017	1:30 pm	675–775	0.5	3.5 km × 7 km	2600
FLEX	To be launched in 2022	10:00 am	500–780	0.3−2.0	0.3 km × 0.3 km	150
TECIS-1	To be launched in 2021	10:30 am	670–780	0.3	2 km × 2 km	34

* The nadir pixel size of MetOp-A Global Ozone Monitoring Experiment 2 (GOME-2) has decreased to 40 km × 40 km since 15 July 2013. ** The spatial resolution of the SCanning Imaging Absorption spectroMeter for Atmospheric CHartography (SCIAMACHY) fitting window for SIF retrieval is 30 km × 240 km, while the footprint size of the nadir mode is approximately 30 km × 60 km.

**Table 2:** Main input parameters for the Soil Canopy Observation Photosynthesis and Energy (SCOPE) model simulations.

Parameter	Description	Value/Range	Unit
N	Leaf thickness parameters	1.4	-
LAI	Leaf area index	0.5, 1, 2, 3, 4	
fqe	Fluorescence quantum yield efficiency at photosystem level	0.01, 0.02, 0.04	-
Cab	Leaf chlorophyll a + b content	5, 10, 20, 40	μg cm^−2^
Cdm	Leaf equivalent water thickness	0.012	g cm^−2^
Cw	Dry matter content	0.009	cm

**Table 3:** Main input parameters for the MODerate resolution atmospheric TRANsmission (MODTRAN 5) model simulations.

Parameter	Description	Value	Units
MODEL	Geographical-seasonal model atmospheres	2, 3	-
H_2_OSTR	Vertical water vapor column	0.5, 1.5, 2.5, 4	g cm^−2^
O_3_STR	Vertical ozone column	0.2	atm-cm
IHAZE	Type of extinction	1	-
VIS	Surface meteorological range	−0.1, −0.2, −0.3, −0.4, −0.5, −0.6	km
H2	Final altitude	0.01, 0.05, 1, 2	km
ANGLE	Initial zenith angle as measured from H1	164, 180	degree
RO	Radius of the earth at the particular latitude	6378.39, 6371.23, 6356.91	km
PARM2	Solar zenith angle at H1	15, 30, 45, 70	degree
V1	Initial frequency (as a wavenumber)	12,500	cm^−1^
V2	Final frequency	16,667	cm^−1^

**Table 4:** Several simulated experiments with different combinations of SR and SNR. ‘No’ in the SNR column indicates that the simulated data do not include noise signals.

Experiment	No.	SR (nm)	SNR
Exp I	1	0.1	127
	2	0.3	322
	3	0.5	472
Exp II	4	0.1	No
	5	0.3	No
	6	0.5	No
	7	0.1	322
	8	0.5	322
Exp III	9	0.3	450
Exp IV	10	0.1	50, 80, 100, 127, 150, 200
	11	0.3	200, 250, 300, 322, 350, 400, 450, 500

**Table 5:** Statistical parameters for the comparisons between the retrieved and true fluorescence derived from the simulated dataset with different combinations of SNR and SR. The statistical parameters include the root-mean-squared difference (RMS diff), correlation coefficient (r), standard deviation (σ), bias between the retrieved and true fluorescence, slope and intercept of the linear fit, and the RMS difference after correction for systematic errors (RMS diff*). ‘No’ in the SNR column indicates that the simulated data do not include noise signals. The units used in columns with bold headings are mW m^−2^ sr^−1^ nm^−1^. In addition, the bold rows represent the best statistical results for simulations with an SR of 0.1 nm and SNR of 127 and those with current specifications (SR of 0.3 nm and SNR of 322) of SIFIS at both far-red and red bands.

Line	Band	SR (nm)	SNR	RMS diff	r	σ	Slope	Bias	Intercept	RMS diff*
1	Far-red	0.1	No	0.66	1.00	1.43	0.79	−0.04	−0.05	0.03
2		0.3	No	0.69	1.00	1.46	0.81	−0.11	−0.14	0.07
3		0.5	No	0.73	1.00	1.48	0.82	−0.18	−0.23	0.12
**4**		**0.1**	**127**	**0.64**	**1.00**	**1.45**	**0.80**	**−0.02**	**−0.03**	**0.15**
**5**		**0.3**	**322**	**0.69**	**0.99**	**1.47**	**0.81**	**−0.10**	**−0.13**	**0.20**
6		0.5	472	0.74	0.99	1.50	0.82	−0.18	−0.22	0.26
7		0.1	322	0.66	1.00	1.44	0.80	−0.04	−0.05	0.07
8		0.5	322	0.76	0.98	1.52	0.83	−0.18	−0.21	0.35
9		0.3	450	0.49	0.99	1.50	0.82	0.12	0.14	0.17
10	Red	0.1	No	0.35	1.00	0.49	0.73	−0.03	−0.04	0.04
11		0.3	No	0.38	0.99	0.49	0.73	−0.05	−0.07	0.07
12		0.5	No	0.56	0.93	0.47	0.67	−0.17	−0.25	0.18
**13**		**0.1**	**127**	**0.49**	**0.84**	**0.58**	**0.73**	**−0.04**	**−0.05**	**0.43**
**14**		**0.3**	**322**	**0.60**	**0.73**	**0.65**	**0.71**	**−0.07**	**−0.08**	**0.62**
15		0.5	472	0.96	0.49	0.94	0.69	−0.13	−0.11	1.30
16		0.1	322	0.37	0.97	0.51	0.74	−0.03	−0.03	0.18
17		0.5	322	1.59	0.27	1.48	0.61	−0.25	−0.34	5.61
18		0.3	450	0.54	0.82	0.57	0.70	−0.08	−0.10	0.47
